# The Holy Grail: highlighting the need for equitable access to dementia treatments and clinical trials

**DOI:** 10.1016/j.lanwpc.2025.101492

**Published:** 2025-02-08

**Authors:** Rebekah M. Ahmed, Olivier Piguet, Catherine J. Mummery, Sharon L. Naismith, Muireann Irish

**Affiliations:** a Department of Neurology, Royal Prince Alfred Hospital, Sydney, Australia; b Brain and Mind Centre, University of Sydney, Australia; c School of Psychology, The University of Sydney, Australia; d Dementia Research Centre, Department of Neurodegenerative Disease, UCL Queen Square Institute of Neurology, University College London, London, WC1N 3AR, UK

**Keywords:** Dementia, Alzheimer's disease, Mono-clonal antibodies, Treatment, Frototemporal dementia

## Abstract

In the last 5 years significant progress has been made in potential dementia treatments, yet many of these treatments come with significant burdens on the healthcare system that may limit access to treatment and care for patients. Often patients in remote and rural regions and those in low income regions are disadvantaged. Many clinical trials for dementia patients are biased to recruiting a homogenous group of patients that does not represent cultural and linguistic diversity, meaning the generalisability of trials is limited. This viewpoint discusses the barriers to access to early treatments and clinical trials for patients with dementia and offers a potential framework to address these including provision of infrastructure, regulatory change and patient education.

The global prevalence of all neurodegenerative dementias is forecast to rise rapidly over the coming decades. The largest rises will be in low to middle income countries (LMIC), reflecting an absolute increase in the population and rapid growth in older populations in these countries. High income countries (HIC) are not immune, from an overall increase in dementia prevalence, and Indigenous and rural populations within these countries will face a disproportionate burden of dementia.^1^

Direct medical care accounts for approximately 20% of the estimated costs of dementia, with the additional economic impact reflecting the indirect costs that stem from informal and social care (unpaid family care or residential care).^2^ Much of the current recorded cost of dementia care is incurred by high income countries, which have a lower comparative prevalence of dementia.^3^ The expected costs of dementia are projected to rapidly and disproportionately increase in countries with emerging economies and large populations.^2^ Given these increased costs, there is an urgent need for equitable access to treatments that may slow or cure these conditions.

Despite the pressing social and economic imperative, new pharmacological treatments for dementia have been slow to develop with only one new drug (Aducanumab) coming to market in the past 20 years, which was subsequently withdrawn by the pharmaceutical company due to financial priorities. Recently, two new candidates, Lecanemab and Donanemab, have emerged, whilst these treatments have been approved in most countries, they are the subject of varied responses globally in terms of funding. The predominant clinical sentiment is that while these treatments are promising, they represent just one piece of the puzzle in terms of a global response to dementia, with major issues surrounding access to available treatments, non-pharmacological options for post-diagnostic support and clinical trial development for new therapeutics. Moreover, whilst the impetus of research has focused on Alzheimer's disease, this has taken the focus away from other dementia syndromes, many of which are just as burdensome in terms of social and economic costs, as well as impact on the individual and caregivers.^4^ The current viewpoint aims to review some of the barriers preventing access to new treatments for dementia including financial, healthcare system and patient education, and proposes a framework to overcome these difficulties. The review also discusses current issues with clinical trial access and generalisability of trial results.

## 1. Emerging treatments for Alzheimer's disease

### a) Healthcare system financing

Two treatments are currently available to treat Alzheimer's disease: Lecanemab and Donanemab. Lecanemab has approval in the USA, UK, Japan, China, South Korea and Israel, with the European regulatory agency initially rejecting but then recommending approval on appeal. Following just behind, Donanemab currently only has approval in the USA and Japan, with other countries currently considering. In Australia these medications are currently under review by the Therapeutic Goods Administration (TGA), with an initial decision to reject the approval of Lecanemab, but an appeal pending. Monoclonal antibodies have pros and cons to their use, making the decisions about their approval or rejection complex (see Table 1 for summary). The initial rejection in Australia was based on a risk benefit analysis stating that the modest benefits did not outweigh the risks. An obvious question is whether these decisions are influenced by medication cost, and the reluctance of governments to sign up for a medication that could in the long run cost the health system hundreds of million to billions of dollars. It has been estimated, for example, using current models of cost that the use of monoclonal antibodies to treat all eligible patients in Europe per year could potentially cost €133 billion, meaning these treatments are not cost effective,^5^ but these models do not take into account the potential cost savings to the healthcare system and reduction in carer burden by slowing disease progression. Other studies have suggested that the number of eligible patients may vary, with the number of eligible patients with early AD and no complicating factors such as vascular disease being small.^6^^,^^7^ Concern has been expressed regarding how these medications will be approved and the funding models used in their administration, with calls for pricing policies that allow equitable access.^5^ Crucially, these treatments lack financial reimbursement in several countries, meaning ultimately it is the patient who is left out of pocket. In Australia, a similar situation has arisen in the context of rare cancers, whereby despite TGA approval, these medications are not funded on the pharmaceutical benefits scheme. This decision has resulted in patients being compelled to use their life savings to fund available treatments in an effort to prolong their life, in turn raising issues of inequitable access.^8^

**Table 1:** Pros and Cons to Monoclonal antibody use.

Pros	Cons
Potential to slow disease progression by a moderate amount	Expense of drugs including infusions, monitoring, follow-up and scans
Provide hope to dementia patients	Infrastructure required to administer drugs including diagnosis, infusion, imaging, amyloid PET scanning
Provide an opportunity for clinicians, researchers, government and industry to reflect on provision of Dementia care and collaborate to provide better access to services for patients	Significant risk of Amyloid related imaging abnormalities particularly in those who a APOE E4 homozygotes

The absence of financial reimbursement for currently approved dementia treatments raises ethical questions for medical practitioners, as often the evidence for treatment efficacy is modest yet patients and their families are arguably desperate for any intervention, even with modest benefit. This appears to be the case for the monoclonal antibodies available for the treatment of AD. Both drugs demonstrated highly effective clearance of the Amyloid- Beta protein in the brain. Functionally, however, the benefits were much less marked, with both drugs showing a small change on a measure of dementia symptom severity–the Clinical Dementia Rating (CDR) Sum of Boxes score–compared with control groups (Donanemab: −0.6 point; Lecanemab: −0.45 point). In addition, high rates of amyloid-related imaging abnormalities (ARIA) were observed in the treatment groups (Donanemab: 24%; Lecanemab: 12.6%), with 3 deaths observed in the treatment arm.^9^^,^^10^ It should be noted that the majority of ARIA cases were asymptomatic.

Arguably, and despite these mixed results, clinicians are reluctant to withhold these treatments, given the clear appetite for any form of therapeutic that may alleviate clinical symptoms of dementia, and the desire to let patients have an informed choice on the basis of both clinical benefit and potential side effects. At present, no consensus has been reached to guide clinical decision-making in this respect.

### b) Healthcare service delivery and workforce

As the currently available monoclonal antibodies must be delivered by regular infusions, administration of these drugs constitutes a significant challenge, especially in the aftermath of the COVID-19 pandemic. The Australian healthcare system remains under extreme stress with budget restrictions and a shortage of staff trained to administer infusions. A recent survey of Australian clinicians found that while knowledge of monoclonal antibodies was high, only 33% felt confident in administering these treatments. The major barriers reported included lack of real-world experience, lack of models of care, lack of guidelines, difficulty with clinical set-up, and lack of information regarding safety.^11^ If monoclonal antibodies are approved in Australia, a major overhaul in how we diagnose and treat patients with dementia will be needed. Current diagnostic criteria for Alzheimer's disease^12^ emphasise the importance of biomarkers (e.g., CSF, PET imaging, p-tau from blood), yet only 40% of Australian clinicians report using biomarkers in their diagnosis of dementia.^11^ While evidence of underlying amyloid via either CSF or amyloid PET brain imaging is a prerequisite to offering these treatments, these diagnostic methods are only available in metropolitan centres and are not covered by Medicare. Adhering to this biological definition of Alzheimer's disease therefore excludes patients in regional, rural or remote regions as well as those who lack the capacity to attend metropolitan centres. The absence of financial rebate further compounds this issue, as once patients are treated, multiple brain MRIs (4–5 per year, in the first year) are required to monitor for safety, adding further pressure on an already stretched system and significant burden to patients and their families. In addition to multiple MRIs, repeat amyloid PET scans and specialist consultations are required to examine response to treatment, further adding to the financial costs. Such constraints act to limit the availability of treatments to patients in more affluent, urban areas, and widen the gap with those living in disadvantaged or remote regions.^13^

### c) Healthcare information

Patient education is also another major challenge. While promising, these treatments are not the panacea often promoted by the media, and it is essential that patients are educated on the risk–benefit profile of available drugs and their mechanism of action. Crucially, patients must be aware that these drugs are not a cure, and at best may slow disease progression although, as noted, such effects are relatively modest.^14^ Results should be communicated to patients in a understandable way, with many patients not understanding numerical scales, but will understand concepts such as “time saved”. Nuanced and potentially candid discussions are needed between patients, their families and treating team covering the full gamut of treatment options including likely risk profiles, consideration of familial risk and the need for genetic counselling. Such discussions need to consider *APOE* gene testing to ascertain one's treatment risk profile given that *APOE* E4 homozygotes have a significantly increased risk of side effects such as ARIA, when treated using monoclonal antibodies.^9^^,^^10^ Moreover, families need to be aware of the potential repercussion of genetic testing on future generations and their concomitant financial implications (e.g., life insurance). The risk benefits of these drugs should also consider the response differences in patients in relation to tau protein load with the Donanemab study finding patients with a high tau load had a poorer clinical response compared to those with a low tau load.^9^^,^^10^ Expert panel guidelines are required to assist clinicians in patient selection that will ensure patients have the best chance of high treatment response with lowest side effect risk.

As should be apparent, while the monoclonal antibodies are promising, we are just at the beginning of the treatment development journey and these drugs are by no means the complete solution to the Alzheimer's disease epidemic. Indeed, current ‘real-world’ estimates within memory clinics suggest that only 6–8% of patients may be eligible to receive these drugs.^6^^,^^7^^,^^15^ Significant and ongoing investment is required in clinical trial development and other forms of post-diagnostic care and management, not only in the context of Alzheimer's disease but also for the treatment of other neurodegenerative dementias.

## 2. Issues with clinical trial development

### a) Unequal access to clinical trials and generalisability of trial results

Historically, clinical trials have been highly inequitable. For example, reports indicate that over a 21-year period of AD clinical trials, 88% were conducted in high income countries with only 11.6% in low or middle income countries.^16^ This concentration of clinical trial activity in high income countries serves not only to compound the unequal access to trials but has knock-on effects in terms of the generalisation and applicability of trial results given mounting evidence of uneven distribution of risk factors, genetic and racial diversity across geographical and socioeconomic regions.^16^ Trials to date have tended to recruit largely homogenous patient samples comprising white, well-educated and well-resourced patients. Even trials that prioritise recruitment of ethnically diverse populations often fall short of their recruitment targets, with reasons for these failures not clear.^17^ Possible factors include difficulty in providing a study partner, unclear medical criteria with unnecessary restriction due to previous medical conditions, narrow use of cognitive criteria including the fact that the MMSE and Clinical Dementia Rating scale which are routinely used are often not suitable for use in diverse patient populations.^18^ Unequal access to clinical trials is further confounded by a constellation of barriers relating to infrastructure, regulatory hurdles, geographical access, and patient education and awareness.

### b) Infrastructure demands

Clinical trials impose immense infrastructure demands, including trained staff (doctors, nurses, clinical coordinators, research assistants), imaging facilities, pharmacy and administration and support for ethics, governance and contractual arrangements. Multiple trained staff members are needed to run trials and keep up to date with changes in protocols, administering treatment, collecting biomarkers and imaging and ensuring safety and compliance. Given these demands, a vicious cycle of inequity emerges whereby only select centres in affluent areas have the resources, expertise, and organisational support to successfully run these trials. In turn, this leads to further inequity in terms of where funding for clinical trials is directed, with successful centres often securing the lion's share, thereby perpetuating the lack of diversity in terms of clinical trial participation. Moreover, the continuing push to keep patient numbers and length of bed stays in hospitals low is short sighted and overlooks the potential long-term benefits of investing in clinical trial infrastructure in hospitals. Also clinicians and nurses input to clinical trials is often not valued by the system and they are often pulled away due to ever increasing clinical loads and pressures. The lack of focus on clinical trials neglects to consider the myriad of benefits associated with early dementia diagnosis and treatment in specialised memory clinics, including prolonged independent living, improved prognosis and quality of life for the individual as well as being cost-effective.^19^

### c) Patient selection

Pharmaceutical trials are inherently complex and require years of research and development. Clinical trials are likely to have the greatest success if started early in the dementia trajectory, before widespread and irreversible neuronal damage. Many studies have shown that whilst the monoclonal antibodies are very effective at removing amyloid, the resultant cognitive decline is irreversible,^10^ which may be dependent on the disease stage, meaning that acting early has the greatest chance of success in stabilising patients at their current cognitive level. Identifying the individuals most likely to develop Alzheimer's disease requires specialist skills and knowledge and is labour-intensive, particularly in regions where biological markers of the disease are not routinely available. Clinical trials require the enrolment of patients in the early symptomatic phase of the pathological process and often include concerted follow-ups for long periods of time; an immense investment on the part of the patient and clinical centre and one which can lead to patient attrition and expiration of drug regulations and patents during that time.^20^ It has recently been suggested that the fact that clinical trials are a success is truly remarkable and attributable to the multidisciplinary teams and infrastructure that must be sustained over long periods of time.^20^

### d) High income countries versus low to middle income countries

As mentioned previously the majority of clinical trials are focussed in high income countries with low to middle income countries often forgotten. Particular barriers to clinical trial and research development in LMIC countries include an awareness of health research and clinical trials, motivation and training of staff to work in clinical trials, the knowledge and technical skills and training to conduct trials and trial leadership capabilities. Often at an operational and government level there is a lack of regulatory infrastructure, financial assistance and knowledge on how to implement trials and help individual centres to offer cutting edge treatements to patients.^21^ One particular issue in LMICs is cultural and religious differences in approach to medicine and trial participation that often hampers research and trial involvement.^22^

## 3. A principled framework towards equitable drug development and delivery

**Fig. 1:**
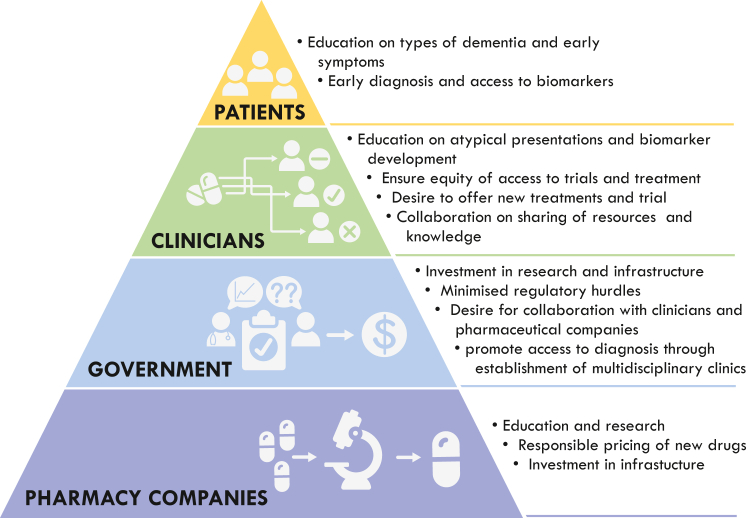
**Framework for improving patient access to Clinical trials and new treatments for dementia**.

This Viewpoint highlights the challenges of ensuring equitable access to early treatments and clinical trials for people living with dementia globally. As can be seen many of these issues are shared between new treatments and clinical trial access and between HIC's and LMIC's, but are further amplified in LMIC's due to lack of development. To address these issues, we advocate for a multifaceted approach [see Fig. 1]. Raising awareness of these issues is the first major hurdle, something that is gradually occurring with the development and potential roll-out of the monoclonal antibodies. As approval of monoclonal antibodies for the treatment of AD is being considered, we anticipate several pressing issues that will need to be addressed including the need for a principled framework to ensure fair and equitable access to the drugs, financial support, as well as health and administrative support. The framework will also need to take into consideration potential geographical challenges to ensure that patients living in regional, rural and remote regions and LMIC's are not overlooked.^23^

### a) Healthcare leadership and governance

Governments will play a vital role in ensuring that pharmaceutical companies bring new treatments to the marketplace at an affordable price for all patients. Ultimately, if treatments are to become globally available, a complete overhaul of the healthcare system will be needed. One model of care that may be leveraged is to provide patients with access to diagnostic biomarkers and shared care models with primary care, as has been successfully deployed in obstetrics to lessen burden on specialist clinics.^24^

Governments further need to invest in the appropriate infrastructure for uniform diagnosis aligned with international standards. Many countries such as Australia have a somewhat haphazard approach to memory clinic organisation, and lack uniform standards for accurate diagnosis and treatment.^25^ For a country as geographically expansive as Australia, significant work is required to implement uniform guidelines across memory clinics,^25^ to ensure that no matter where a patient is located they are guaranteed to receive the same level of care and access to clinical trials and treatment. This issue will be even more pertinent in the western pacific region where some countries (e.g., Indonesia) are dispersed over multiple small islands. Governments need to invest in healthcare systems that ensures every patient is reviewed by adequately trained staff in the diagnoses of dementia, including those with atypical dementias, and have access to biomarkers to ensure early and quick diagnosis that will then flow onto treatment availability. Policy change is required at a government level to allow early access to clinical trials and new treatments, with a critical need to concurrently expand both memory clinic and clinical trial infrastructure capacity. The Australian government has arguably made recent attempts to address some regional inequalities, through targeted programs to help regional patients become involved in clinical trials.^26^ The efficacy of such approaches, however, will likely depend on how well such measures are sustained over time. Further measures also include training staff in diagnosis, and ensuring they have access to the latest imaging and biomarker techniques. Better collaboration is also required with training of site staff to engage in clinical trials. Often the training required to participate in trials is onerous with each pharmaceutical company requiring their own training for widely available outcome measures. Possible ways to alleviate this include centralised training resources that are recognised by multiple companies, and government training models in developing and delivering clinical trials for patients with dementia.

Pharmaceutical companies are also keen for quick turnarounds, which are hampered by long regulatory framework lead–in times including ethics and governance reviews. Regional differences in clinical trial governance can result in gross disparities. For example, a clinical trial may take 6–12 months to gain regulatory approval in one country, while the same trial may be quickly fully operational in other more affluent parts of the world. This results in competitive recruitment whereby a recruitment target is set and trial places are filled quickly by patients in higher-income countries, while patients in less well-resourced countries fail to gain access due to long regulatory delays. Discussions with government and organisations are required to reduce regulatory wait times potentially via the establishment of common ethical and governance approvals and enforced review timelines. This will ensure that trials are approved quickly throughout the world giving patients equal access to enrolment.

### b) Healthcare information and education

Patient education is a further crucial barrier that influences awareness and understanding of clinical trial participation. Patient advocacy groups acknowledge the inherent challenges in communicating the requirements of clinical trials, the relative risk–benefit profiles, and the different stages of drug development to prospective participants. Dedicated community education is required to raise public awareness of the earliest signs of dementia and the importance of obtaining an early diagnosis to have the best possible benefit from treatment. To overcome the disparity in access to clinical trials, relevant literature is needed in multiple languages and provided in a culturally appropriate manner, in partnership with interpreters. Patient education material is required to explain what involvement in a clinical trial involves and what are the pros and cons. Mandatory enforcement by ethics organisations of lay person summaries is required. Other measures could include ethics boards mandating the inclusion of consumers in trial development and education material to ensure that patient and carer voices are heard and that the priorities of researchers and consumers are firmly aligned. Measures to combat cultural differences in the stigma surrounding dementia is also required and trust in healthcare systems to allow patients to come forward for diagnosis and to participate in trials. Efforts are also required by pharmaceutical companies and researcher to include non-English speaking candidates, which in turn will require the development of cognitive measures in multiple languages and sensitive to cultural norms. Such steps are necessary if clinical trials are to be regarded as truly global and representative of the population.

Further resources are required to ensure patients are supported throughout their trial experience, providing practical solutions to overcome financial and transport barriers. One such suggestion to overcome these barriers is the implementation of “dementia navigators” who understand the healthcare system and can help patients navigate not only the clinical aspects of diagnosis and access to services, but also participation in clinical trials.

To date, the discussion surrounding access to new treatments has been restricted to high income economies, with low-to-middle-income countries often overlooked. A concerted effort is required to bridge this gap including collaboration between HIC and LMI countries and governments to train staff and share resources, including both clinical/infrastructure and regulatory guidelines effectively.^21^ Pharmaceutical companies can play an important role in this context by ensuring clinical trials are accessible to people in LMICs. This may involve establishing partnerships across trial centres, so that less resourced LMICs can benefit from the expertise of higher-income experienced trial centres. Examples of such capacity-building initiatives include the FTD Prevention Initiative (FPI), which aims to bring access to clinical trials and research to patients with frontotemporal dementia throughout the world through collaboration between researchers in HIC and LMIC's and pharmaceutical companies. Members of this initiative include countries such as the USA, UK, Australia, India, Singapore, Japan, China and India.^27^

Effort is also required to encourage pharmaceutical companies to collaborate with researchers working on non-Alzheimer dementias to ensure the development and trial of medications for other debilitating neurodegenerative disorders (e.g. frontotemporal dementia, Dementia with Lewy bodies). This could extend to further capacity building initiatives such as targeted funding calls to support partnerships between researchers and industry to develop new treatments. Dementia consumer organisations can play a formative role in educating patients with less common dementias to be involved in research and to seek out clinical trial opportunities. Successful endeavours thus far include education series for example by the Association for Frontotemporal Degeneration with a strong focus on clinical trial and treatment development.

Governments, pharmaceutical companies and clinicians need to ensure that equity to treatment access is a priority for dementia patients. This will require changes at local, national and international levels, ranging from patient education and resource provision to clinicians, to governmental provision of adequate infrastructure and regulatory collaboration. Pharmaceutical companies can also play a crucial role by providing special access schemes for medications, appropriate pricing, and viewing their role beyond merely manufacturing medications to that of providing infrastructure, education, health system support, as well as research on a global scale.

We conclude this viewpoint at an exciting juncture in dementia treatment. After many years of publicised treatment failures, there is a genuine belief that we may be at a turning point in being able to offer patients hope. Now is the time for governments, pharmaceutical companies, medical professionals and patients to come together to fight these devastating diseases.

## Contributors

Rebekah M Ahmed: manuscript concept, writing and editing.

Olivier Piguet manuscript writing and editing.

Sharon Naismith manuscript writing and editing.

Catherine Mummery manuscript writing and editing.

Muireann Irish manuscript writing and editing.

## Declaration of interests

RMA has received payment for Eisai, Eli Lilly and Biogen Scientific board membership, CJM has received payment for Eisai, Novartis, Roche, Eli Lilly and Biogen Scientific board membership, SLN has received consulting fees for Eisai and Eli Lilly. None of these payments were related to the current manuscript.
